# A Case of Hypereosinophilic Syndrome Presenting with Multiorgan Thromboses Associated with Intestinal Obstruction

**DOI:** 10.4274/Tjh.2012.0141

**Published:** 2013-09-05

**Authors:** Tao SUI, Qing Li, Li Geng, Xinnv Xu, Yuming Li

**Affiliations:** 1 Department of Hematology, Tianjin First Center Hospital, Tianjin, China; 2 Key Lab for Critical Care Medicine of the Ministry of Health, Tianjin First Center Hospital, Tianjin, China; 3 This author contributed equally to this work and should be considered as co-first author

**Keywords:** Eosinophilia, Hypereosinophilic syndrome, Thrombosis, Intestinal obstruction

## Abstract

Idiopathic hypereosinophilic syndrome (HES) is a disease characterized by persistent hypereosinophilia (>1.5×109/L) for more than 6 months in the absence of other causes of reactive eosinophilia. Patients with HES presenting with multiorgan thromboses are rare. Herein we report a 57-year-old man with HES who presented with deep venous thrombosis of the lower extremities, portal thrombosis, pulmonary embolism, and mesenteric venous thrombosis, which led to intestinal obstruction.

**Conflict of interest:**None declared.

## Abstract (tr)

İdiyopatik hipereozinofilik sendrom (HES) reaktif eozinofilinin diğer nedenlerinin yokluğunda 6 aydan uzun süren persistan eozinofili (>1.5×109/L) ile karakterize bir hastalıktır. Multiorgan trombozları ile başvuran HES’li olgular nadirdir. Burada, alt ekstremite derin ven trombozu, portal tromboz, pulmoner embolizm, ve özellikle intestinal tıkanıklığa yol açan mezenterik venöz tromboz ile başvuran 57 yaşında bir erkek olgu sunulmuştur.

## INTRODUCTION

Idiopathic hypereosinophilic syndromes (HESs) are rare disorders that comprise a heterogeneous group of diseases characterized by unexplained persistent, non-reactive overproduction of eosinophils (>1.5×109/L) persistent for more than 6 months. HES can cause multiple organ damage/dysfunction, mainly involving the skin, heart, lung, gastrointestinal tract, and nervous system [1]. The clinical manifestations of HES include fever, rash, fatigue, cough, shortness of breath, muscle aches, and diarrhea. The most common presenting manifestation is thrombosis, such as mural thrombus of the heart, inferior vena cava thrombosis, superficial venous thrombosis, portal thrombosis, deep venous thrombosis, cerebral arteriolar and venous thrombosis, and intracardiac thrombi [2,3,4,5]. Simultaneous multiple organ thromboses associated with HES rare. Herein, we report a unique case of HES presenting with deep venous thrombosis of the lower extremities, portal thrombosis, pulmonary embolism, and especially mesenteric venous thromboembolism, which led to intestinal obstruction. 

## CASE REPORT

A 57-year-old man was admitted to our department for pain and swelling of both lower extremities accompanied with fever for more than 10 days. His medical and family histories were unrevealing. His vital signs were within the normal range and the clinical examination was normal, except for hyperemia, warmth, slight tenderness, and edema of both lower legs, especially in the right leg. White blood cell count was high (23.9×10^9^/L) with 66.5% eosinophils (15.9 ×10_9_/L), hemoglobin of 141 g/L, and thrombocytopenia of 11×10^9^/L. Liver and renal function tests were normal. A test for parasites in the stool and serum was negative. Tests for tumor markers were negative. The work-up for allergic and rheumatologic diseases were also negative (including anti-nuclear, anti-dsDNA, anti-neutrophilic cytoplasmic antibodies; anti-phospholipid antibodies; anti-SSA, anti-SSB, anti-histone antibodies). A bone marrow aspiration and biopsy were performed and hematological malignancies were excluded. The bone marrow aspiration revealed a significant proliferation of trilineage cells and a prominent component of mature-appearing eosinophils comprising 68% of the total cellularity. The bone marrow biopsy showed normal cellularity with increased eosinophils. Polymerase chain reaction indicated a negative BCR-ABL while fluorescence in situ hybridization indicated a negative FIP1L1-PDGFRA gene fusion. An ultrasound confirmed the arteriosclerosis of both lower limbs accompanied by multiple plaques and thrombosis of the right popliteal vein. An abdominal ultrasound revealed fatty liver and thrombosis of the portal vein. Echocardiography showed that the patient’s atrioventricular cavity size, ventricular wall thickness, range of motion, and valve morphology were all normal. Ejection fraction was 69%.

**Figure 1:**
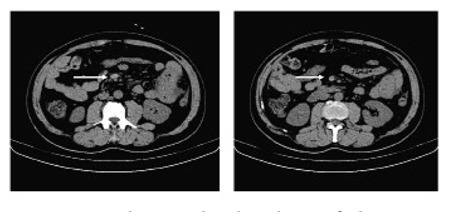
CT showing the thrombosis of the superiormesenteric vein (arrow).

**Figure 2:**
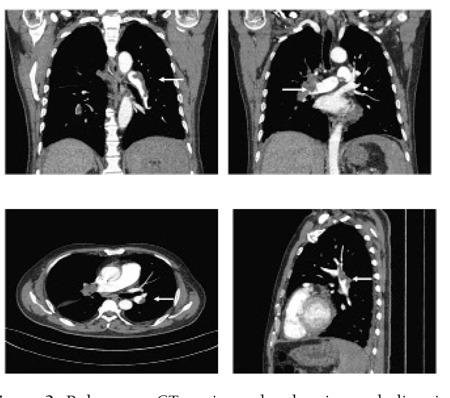
Pulmonary CT angiography showing embolism inthe pulmonary arteries and their branches (arrow)

At this stage, HES with multiorgan thromboses was suspected. Methylprednisolone (40 mg/day) was commenced and piperacillin was initiated, empirically. Hemorrhagic spots appeared on the skin of both lower limbs, and blood blisters of the oral mucosa were observed at a certain period. Thus, platelets were transfused.Pain and swelling of both lower extremities were alleviated using the aforementioned treatment. At day 10, abdominal distension and abdominal pain occurred and the abdominal pain could not be relieved by anisodamine hydrochloride. It gradually worsened and abdominal tenderness emerged with rebound tenderness as well as muscle tension. A plain abdominal radiograph revealed intestinal obstruction. No neurologic abnormalities were noted. White blood cell count was 8.07×10^9^/L with 26.2% eosinophils (2.12×10^9^/L), 10^7^ g/L hemoglobin, and 18×109/L platelets. No evident presence of fragmented red blood cells was observed in the peripheral smear. The renal function was normal and The prothrombin time (PT) was 18.2 s and the activated partial thromboplastin time (aPTT) was 40.9 s. Fibrinogen was 8.1 g/L and D-dimer was 9.6 µg/mL. Computed tomography (CT) results suggested that for the thrombosis of the superior mesenteric vein (Figure 1), emergency partial excision of the small intestine could be performed. The histopathological investigation confirmed the extensive small bowel hemorrhage, necrosis, and mesenteric vascular thrombosis. Postoperative recovery was good. Continued treatment with glucocorticoid and anticoagulant therapy was not performed due to the patient’s very low platelet counts with higher risk of bleeding. On day 29, the patient had dyspnea and breath-holding accompanied by chest pain and intermittent expectoration with bloody streaks. Oxygen saturation was 85% without oxygen support. Auscultation of the lungs revealed moist rales. Pulmonary CT angiography showed embolism in the bilateral pulmonary arteries and their branches, as well as bilateral pleural effusion 

(Figure 2). Meanwhile, protein C and S levels were normal.

An ultrasound of the abdomen and both lower limbs showed that the thrombosis of the right popliteal vein and portal vein had no significant alteration compared with previous images. The white blood cell count was 8.69×10^9^/L, with 31.3% eosinophils (2.72×10^9^/L), 90 g/L hemoglobin, and 21×109/L platelets. The PT was 17.9 s and the APTT was 57.6 s. Fibrinogen was 2.43 g/L and D-dimer was 2.6 µg/mL. Heparin (2000 IU/day) was initiated. Platelets were transfused when the counts were lower than 10 × 109/L. On day 35, the patient’s eosinophil count returned to normal; the white blood cell count was 6.68 × 10^9^/L with 0.2% eosinophils (0.02×10^9^/L), hemoglobin of 90 g/L, and platelets of 19 × 10^9^/L. The patient’s condition also gradually stabilized. CT showed that the pulmonary embolism was significantly improved in compared with the previous images. Prednisone (28 mg/day) and coumadin (2.5 mg/day) were initiated. Thus far, follow-up has been consistent; the eosinophil and platelet counts were within normal limits and no recurrence of thrombosis was observed. Informed consent was obtained.

## DISCUSSION

HES is a rare hematological disorder, and its diagnosis is set when patients have persistently elevated eosinophil counts of >1.5 × 10^9^/L for more than 6 months and other causes of reactive eosinophilia have been excluded through histopathological evaluation and imaging studies for internal organ involvement.

Our patient was FIP1L1-PDGFRA-negative and did not show any evidence of T or B cell clonality. The diagnosis of idiopathic HES was considered after asthma, eczema, parasitic infestation, autoimmune disorders, and malignant neoplasm were ruled out. However, hereditary thrombophilia cannot be excluded due to the fact that hereditary factors such as factor V Leiden mutation, prothrombin G20210 mutation, and AT deficiency were not investigated. To the best of our knowledge, multiorgan thromboses, as a presentation of HES especially associated with intestinal obstruction, is rare and have only been reported by Kobayashi et al. [6]. 

When our patient presented with venous thrombosis of both legs, the portal vein, and the superior mesenteric vein, anticoagulation was not performed due to the severe thrombocytopenia accompanied with mucosal bleeding. Glucocorticoids and platelet transfusions were preferred. The eosinophil counts gradually decreased, suggesting that our patient exhibited response to glucocorticoids. However, unfortunately the patient developed pulmonary embolism. Initially, we assumed that the embolism was related to thrombus shedding, and so an ultrasound was performed again. The images showed that the thrombosis of the right popliteal vein and the portal vein had no significant alteration compared to previous images. Pulmonary CT angiography showed multiple embolism in the pulmonary arteries. Thus, the pulmonary embolism may have been due to local eosinophilic infiltration that resulted in hypercoagulability. Therefore, heparin was used for anticoagulant therapy with intermittent platelet transfusions. To date, no other thrombosis has occurred and the platelets generally reached the normal range.

Glucocorticoids are the first-line therapy for FIP1L1-PDGFRA-negative HES and are very effective for decreasing eosinophil counts [7]. INF-α, hydroxyurea, and anti-IL-5 monoclonal antibodies are considered in case of glucocorticoid resistance. In our case, eosinophil counts decreased gradually with glucocorticoids. However, despite the decrease in the eosinophil counts, the risk for thrombosis recurrence did not disappear as reflected by an intestinal obstruction which may have been related to the thrombosis of the mesenteric vein. Although our patient’s eosinophil counts were significantly reduced by glucocorticoid administration, the patient needed emergency operation for the intestinal obstruction. Eosinophil counts do not always seem tomatch with organ damage.

The link between eosinophilia and the thrombotic event is still unclear. One possible reason is the infiltration of eosinophils in involved tissues [8,9,10]. A number of cytotoxic substances are then released, including highly cationic molecules such as eosinophil cationic protein, major basic protein, ribonuclease eosinophil-derived neurotoxin, oxidizing molecules such as eosinophil peroxidase and free oxygen radicals, and enzymes such as elastase and collagenase. Eosinophils can also produce lipid mediators such as leukotrienes and prostaglandins. Another reason is the presence of predispositions to thrombosis, such as immobility, genetic hypercoagulability, or advanced atherosclerosis [4,11,12].

In conclusion, HES with thromboembolic events in multiple organs is usually difficult to manage. Therefore, patients should be monitored carefully for the potential complications of the disease, and treatment should be performed according to the situation of each individual patient.

**Acknowledgments**

This work was supported by the Tianjin Science and Technology Committee (11JCZDJC18600).

## CONFLICT OF INTEREST STATEMENT

The authors of this paper have no conflicts of interest, including specific financial interests, relationships, and/ or affiliations relevant to the subject matter or materials included.
